# Lack of knowledge on ecological determinants and cryptic lifestyles hinder our understanding of *Terfezia* diversity

**DOI:** 10.3897/mycokeys.84.71372

**Published:** 2021-10-18

**Authors:** Celeste Santos-Silva, Rogério Louro, Bruno Natário, Tânia Nobre

**Affiliations:** 1 Biology Department, Macromycology Laboratory, MED - Mediterranean Institute for Agriculture, Environment and Development, University of Évora, Évora, Portugal University of Évora Évora Portugal; 2 MED–Mediterranean Institute for Agriculture, Environment and Development, University of Évora, 7000-083 Évora, Portugal University of Évora Évora Portugal

**Keywords:** Desert truffles, host plants, phylogeny, soil properties, taxonomy

## Abstract

Developing below the soil surface desert, truffles are hard to find. Within *Terfezia* genus, at least 18 species are described and many are endemic to the Mediterranean basin. Ecological and geographic information are key factors for species diagnosis, and so far *Terfezia* species are believed to be linked to either acidic or basic soils or to specific plant hosts. Thus, we have looked at *Terfezia* diversity within a relatively homogeneous geographical area in Portugal that is suitable for these species and that covered different soils and different dominant host species. We analyzed the observed intraspecific variability within the context of species ecological preferences (e. g. edaphic and putative host). One of our major findings was the discovery of *T.grisea* in acid soils in association with *Tuberariaguttata*, a puzzling information since, until now, this species was only found in alkaline soils. We also report on the linkage of different *Terfezia* lineages within species and ecologic parameters such as soil texture, soil pH and plant host. Additionally, by placing the collected specimens on the most recent genus phylogeny based on the ITS region, we also updated the number of known *Terfezia* species occurring in Portugal from three to ten. *Terfeziadunensis* is here reported for the first time for Portugal. Overall, our results show that the exploration of undersampled sites reveals itself as a good strategy to disclose unknown aspects of desert truffle diversity and ecology. These aspects are of prime importance when considering the economic value of the desert truffles for rural populations in the Mediterranean basin.

## Introduction

Desert truffles produce macroscopic fruitbodies partially or completely embedded in soil. These hypogeous *Ascomycota* encompass several genera within the *Pezizaceae* family. *Terfezia* Tul. & Tul. is the most diverse genera of desert truffle with 18 species described, typically found in arid and semi-arid areas throughout the world (Morte et al. 2009; Navarro-Ródenas et al. 2011; Moreno et al. 2014; Louro et al. 2019). Many of the *Terfezia* spp. are endemic to the Mediterranean basin and they play an essential role in soil conservation – preventing erosion and desertification – of Mediterranean shrublands and xerophytic grasslands (Honrubia et al. 1992).

The interest in understanding diversity and the molecular phylogeny of fungi, in particular of desert truffles, has increased in recent years following up from the increasing importance of biotechnology and plant nutrition. In addition, and for *Terfezia*, the interest is even higher as the demand for ascocarp availability/production increased. *Terfezia* products are continuously gaining in relevance as exquisite components of the Mediterranean diet.

Early attempts at *Terfezia* classification relied on morphological characteristics, such as spore and peridium morphology, gleba colour, and chemical features (Bordallo and Rodriguez 2014). Yet, these features alone showed to be problematic to distinguish species. In many hypogeous genera, *Terfezia* included, the evolution for mycophagy and reduction of water loss translated in convergent morphological characteristics and homoplasy (Thiers 1984; Bruns et al. 1989; Diez et al. 2002). The result was an array of species names in which many were synonyms of previously described ones (Alsheikh 1994) and others were lacking useful diagnostic features or were rarely cited after the first time (Zitouni-Haouar et al. 2018). With advances in molecular technology scientists have re-examined herbarium specimens and personal collections of *Terfezia* around the world for their sequences of the Internal transcribed spacer (ITS), the primary fungal barcode. These efforts have revealed inaccurate generic assignments, misidentifications at the genus and species level and, overall, were able to remove ambiguity around several taxonomic statuses involving this genus (Zitouni-Haouar et al. 2018; Louro et al. 2019). This given clarity was not without its inherent difficulties.

The first step in linking diversity to its geographic and ecological determinants is to know the diversity that we are dealing with. Considered as separated species in pre-molecular era, *Terfezialeptoderma* (Tul. & C. Tul.) Tul. & C. Tul. and *T.fanfani* Mattir. are now regarded as one taxa (*T.fanfani*) since phylogenetic studies show a clear nesting of these species sequences in a well-supported monophyletic group (Bordallo et al. 2013, 2015; Louro et al. 2019). Furthermore, *T.leptoderma* and *Terfeziaolbiensis* (Tul. & C. Tul.) Sacc. were by some authors regarded as the same species, being that *T.olbiensis* was considered an immature stage of *T.leptoderma* (Moreno et al. 1986; Diez et al. 2002; Bordallo et al. 2013). Recent studies, however, propose *T.olbiensis* as a unique taxa and absolved *T.leptoderma* from the previously assigned sequences (Montecchi and Sarasini 2000; Louro et al. 2019), with the exception of one sequence (GenBank AF396864) that remains unassigned (Louro et al. 2019, 2020). The spiny spored *Terfezia* complex harbors further phylogenetic difficulties, for example *T.cistophila* Ant. Rodr., Bordallo, Kaounas, & Morte was suggested as a later synonym of *Terfeziatrappei* (R. Galán & G. Moreno) A. Paz & Lavoise (Paz et al. 2017), after suffering a taxonomic change at the genus level from *Elaphomyces* Nees to *Terfezia* (Paz et al. 2017). Later, we showed that *T.cistophila* and *T.trappei* formed two distinct and well-supported clades (Louro et al. 2019). The sequences describing *T.trappei* were recently re-considered as either *T.fanfani* (Vizzini et al. 2019) or as the newly described *Terfeziasolaris-libera* Louro, Nobre, Santos-Silva (Louro et al. 2020) suggesting that *T.trappei* might not be a valid taxon.

Despite all the above contributions, the genus *Terfezia* is still undergoing frequent taxonomic revaluations. It now seems clear that combined efforts are needed: classic taxonomy, molecular biology and ecology have to be worked synergistically. The lack of available sequences regarding the most cryptic species and the lack of a clear description of its ecological and geographic preferences are still obstacles hindering our understanding of the genus diversity.

As with all other truffles, *Terfezia* species are obligate symbionts of specific host plants, mainly members of the *Cistaceae* (Alsheikh 1994; Morte et al. 2009) including different annual and perennial species of the genus *Helianthemum* and *Cistus*, but also with members of the *Fagaceae* and *Pinaceae* (i.e. oaks and pines) (Alsheikh 1994; Diez et al. 2002; Kagan-Zur and Roth-Bejerano 2008; Morte et al. 2009). These plants and their associated *Terfezia* can be found in soils ranging from acidic to basic in their characteristics (Gutiérrez et al. 2003; Morte and Andrino 2014; Bordallo et al. 2015; Dafri and Beddiar 2017). Given their symbiotic nature, host specialization and edaphic tolerances have been hypothesized to have played significant roles in *Terfezia* adaptive evolution (Diez et al. 2002). Therefore, ecologic and geographic information are indisputably key factors for *Terfezia* species diagnosis; many species are thought to occur only in acidic or basic soils or in association with specific host plants (Gutiérrez et al. 2003; Morte and Andrino 2014; Bordallo et al. 2015; Dafri and Beddiar 2017). It is surprising that little to no geographic and ecological information is available for many of the deposited sequences of *Terfezia* in the most popular nucleotide databases. Even when that information does exist, it often seems incongruent, leading to worrying misidentification errors when crossing molecular analysis and ecological information. This seems to be the case with a sequence of an uncultured *Pezizaceae* (GenBank FJ013087) supposedly associated with *Pinuspinaster* Aiton, which corresponds to *T.cistophila* according to the phylogenetic reconstitution from Louro et al. (2019). However, this last finding opposes the initial description that *T.cistophila* lives solely associated with *Cistus* spp. (Bordallo et al. 2015). Another example (discussed in Louro et al. 2019) refers to two sequences given as *T.olbiensis* and associated to *Tuberariaguttata* (L.) Fourr. as putative host plant. *T.olbiensis* is by all accounts associated with *Pinus* spp. and *Quercus* spp. (Bordallo et al. 2013), and the published sequences nest inside *Terfeziaalbida* Ant. Rodr., Muñoz-Mohedano & Bordallo clade (Louro et al. 2019).

At this point it seems that only through a multidisciplinary approach encompassing molecular, morphological and ecological features will we be able to broaden our understanding of *Terfezia* diversity. This especially applies in undersampled regions where the probability of discovering new species is favored due to the cryptic lifestyle of *Terfezia*. Adhering to these these stipulations, we have developed a case study in Portugal, where until 2018 *Terfezia* richness was greatly overlooked, with only three species documented. Since then, five more *Terfezia* species have been recorded *T.cistophila*, *T.extremadurensis*, *T.lusitanica*, *T.pini* and *T.solaris-libera* (Bordallo et al. 2018; Louro 2020; Louro et al. 2020, 2021) and the soil main features and putative host plant were registered. In the present work we reassess the diversity of this genus and characterize *Terfezia* ecology within the framework of ecological preferences, while also probing the intraspecific variability of the *Terfezia* taxa in analysis.

## Methods

### Surveys

The sampling took place between 2013 and 2020 from February to June, in the most favorable months for desert truffle growth. The surveys occurred within the framework of two projects (Santos-Silva 2015, 2020) aiming to develop the technology necessary to produce the two most economically important desert truffles in Portugal, namely, *Terfeziaarenaria* (Moris) Trappe and *T.fanfani*. The specimens were collected in a wide range of habitats within the relatively homogeneous geographical area that is favorable to *Terfezia*. Hence, several areas with documented occurrence of desert truffle were surveyed and all the desert truffle specimens encountered were collected. The putative plant host was registered. Soil samples (50 mm diameter, 150 mm depth) were collected in each sampling site. A compose sample of 6 soil samples replicas per site was analyzed at the Laboratório Químico Agrícola Rebelo da Silva (INIAV/LQARS) for particle size and subsequent soil textural classification and water pH measurements. Throughout the collection period, the fresh ascocarps were brought to the laboratory for morphological and molecular characterization. Fragments of each specimen were frozen at -20 °C for DNA amplification and the remaining specimens were dried at 40 °C and stored in sealed plastic bags, labeled with collection details. All samples are deposited at the Herbarium of the Évora University Herbarium (UEVH-FUNGI), Portugal.

### ITS sequences

DNA extraction from the analyzed specimens was performed by CTAB method, following the protocol described in Nobre et al. (2018). All extraction products were stored at -20 °C and later used directly in the PCR. The Internal Transcribed Spacer (ITS) region of the rDNA, including the 5.8S ribosomal gene, was amplified using the ITS5 and ITS4 primers (White et al. 1990). PCR reactions were conducted using 1 μl of the extracted DNA in a standard 25 μl reaction, with 0.5 pmol/μl of each primer, 1.5 mM MgCl_2_, 0.5 mM dNTPs and 0.04 U/ml Taq DNA polymerase. PCR reactions were performed using a Mastercycler Gradient thermocycler (Eppendorf, Hamburg, Germany) with the following cycling parameters: an initial denaturalization step for 3 min at 95 °C, followed by 35 cycles consisting of: 30 s at 95 °C, 30 s at 55 °C (annealing temperature), 1 min at 72 °C, and a final extension at 72 °C for 10 min. All the PCR products were purified using the NZYGelpure kit (from NZYTech, Lda) and sequencing was done commercially (STAB VIDA, Lda.).

### Phylogenetic reconstruction

Based on the most recent published phylogenetic reconstruction using UNITE curate sequences (Louro et al. 2019) we have selected 42 sequences covering each of the well supported clades. The same three known non-*Terfezia* sequences were selected as putative outgroups: *Tirmania* Chatin (GenBank JF908769.1), *Cazia* Trappe (GenBank AY830852.1) and *Peziza* Dill. Ex Fr. (GenBank JX414200.1). These sequences were aligned with the dataset of newly generated sequences from this work (216 sequences), using the E-INS-i strategy of the online MAFFT version 7 (Katoh et al. 2017). The phylogenetic reconstruction analysis based on the above sequences was performed in BEAST v.4.2.8 software (Drummond and Rambaut 2007), allowing the software to estimate the evolutionary model. All other settings were left as default. The output of BEAST was analyzed in the software Tracer v.1.6 to determine chain convergence and burnin. Trees were combined using the software TreeAnnotator v.2.4.8 to produce the single tree that best represents the posterior distribution, considering a burn-in of 10% (first 1000 trees were removed).

## Results

**Figure 1.**
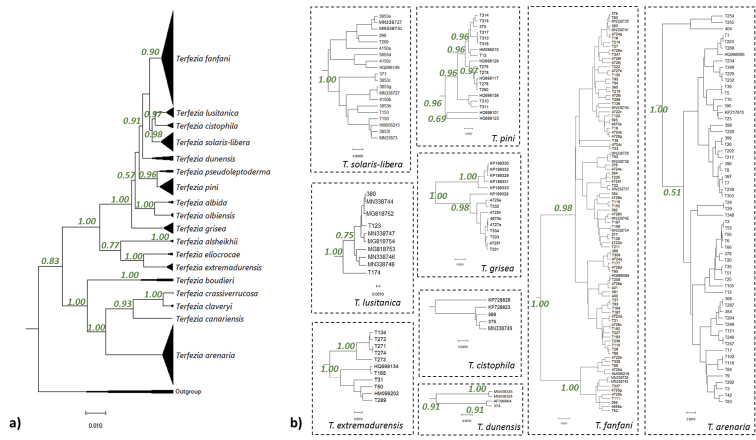
**a** Phylogenetic relationship between *Terfezia* species. The reconstructed phylogeny corresponds to the majority rule consensus tree higher than 0.50 of trees sampled in a Bayesian analysis, and the posterior probability values are shown for main nodes **b** clades with new sequenced specimens collected within the present study.

An ITS amplified fragment with gaps of 721 bp was aligned, comprising 67 bp of the partial sequence of the 18S ribosomal RNA gene; 228 bp internal transcribed spacer 1; 156 bp of the 5.8S ribosomal RNA gene; 221 bp of the internal transcribed spacer 2; and 49 bp of the 28S ribosomal RNA gene. The reconstructed phylogeny ample supports the existence of 18 distinct clades representing well supported monophyletic groups (Fig. 1). Concerning the position of the newly collected specimens, the phylogenetic analysis successfully assigned them to 9 separate clades, namely to *T.arenaria*, *T.cistophila*, *T.dunensis*, *T.extremadurensis*, *T.fanfani*, *T.grisea*, *T.lusitanica*, *T.pini* and *T.solaris-libera* clades. Overall, the total number of registered *Terfezia* in Portugal expanded to 10 species.

Concerning species distribution and representativeness, *T.arenaria* and *T.fanfani* were the most widespread and commonly found *Terfezia* species, being in abundance at every sampling site. All other 7 species seemed to have narrower distribution ranges, however, their stochastic appearance throughout the sampling period made it impossible to confirm their distribution and fructification patterns.

Regarding soil texture, *Terfezia* species occupied areas dominated by loamy sand soils (lSs) (51%) or sandy loam soils (sLs) (42%), and less frequently pure sandy soils (Ss) (7%). As to the soil pH, values varied from 5.1 to 7.3, with 5.6 the most frequent value, and half of the areas sampled showed pH values between 5.6 and 6.0. In other words, the sampled *Terfezia* specimens occupy strongly acidic to neutral soils, ranging from sandy to loamy soils (Table 1, Suppl. material 1: Table S1).

**Table 1.** *Terfezia* preferences relating to host plant and soil (see more details in Suppl. material 1: Table S1). *Tuberariaguttata* – Tg; *Cistussalviifolius* – Cs; *Cistusladanifer* – Cl; *Quercus* spp. – Q; *Pinus* spp – P.

Species	Host plant	Soil type	Soil pH
*T.arenaria*	Tg	Loamy sand, Sandy loam	5.2–7.3
*T.cistophila*	Cs, Cl	Loamy sand	5.5–5.6
*T.dunensis*	Cs, P	Loamy sand	6.1
*T.extremadurensis*	Tg	Sandy loam	5.3–6.0
*T.fanfani*	Tg	Loamy sand, Sandy loam, Sandy	5.1–6.4
*T.grisea*	Tg	Loamy sand, Sandy	5.7–6.1
*T.lusitanica*	Tg	Loamy sand, Sandy	5.5–6.2
*T.pini*	Q, P	Sandy loam	5.3–6.0
*T.solaris-libera*	Tg	Sandy loam	6.0

Despite the observed spatial heterogeneity of the different sampling sites, and the multiple putative plant hosts available, which in some sites included annual plants, *Cistus* shrubs and either *Quercus* or *Pinus* trees, the most frequent putative plant host was *Tuberariaguttata* (91%) (Suppl. material 1: Table S1).

**Figure 2.**
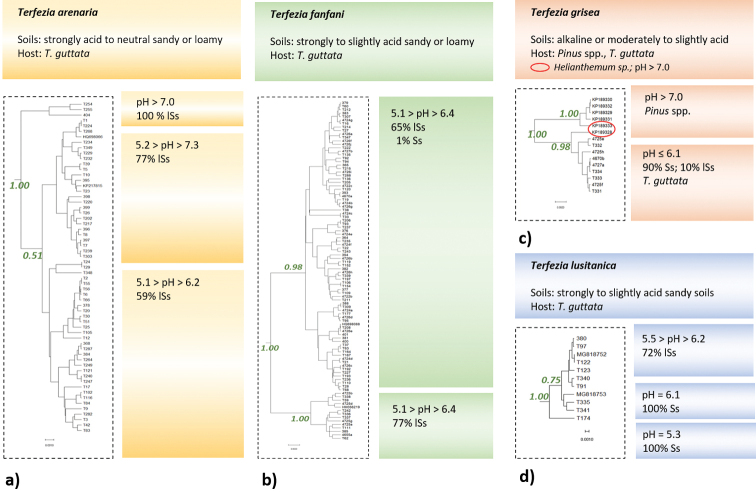
Phylogenetic reconstruction of intra-species diversity (Fig. 1) linking to soil properties and putative host plant **a***T.arenaria***b***T.fanfani***c***T.grisea* [specimens in the circle represent deviations from the ecological grouping, see text for details] **d***T.lusitanica*. The other species are identified and their relation to soil and host plant are presented in the main text.

While checking for possible relations between the specimen’s position in the reconstructed phylogenetic tree and the recorded ecological parameters, we found that proximity of sampling locations was not an influencing factor to explain the multiple lineages (i.e. subgroups) seen within each clade, since specimens from different locations were often grouped together in almost all the subgroups of a given clade. For instance, *T.pini* intraspecific variability, as shown by well supported branches in the reconstructed phylogeny (Fig. 1), comprises specimens collected in Spain and different locations in Portugal in each subgroup. On the other hand, some patterns and tendencies were observed between different *Terfezia* lineages within each clade and ecologic parameters such as soil texture, soil pH and putative plant host (Table 1, Suppl. material 1: Table S1, Fig. 2).

*T.arenaria* occupies strongly acid to neutral sandy or loamy soils and its putative host is only *T.guttata*. In *T.arenaria* intraspecific reconstructed phylogenetic variability (Fig. 1) three groups were formed which seem to show, from top to bottom, a decrease in preference for more neutral and sandier soils. Represented at the top, a small group separates from the others, and these specimens were all collected in lSs with pH higher than 7.0. The second group shows, on average, different preferences to the third, with 77% collected in lSs (pH from 5.2 to 7.3) against 59% lSs (pH from 5.1 to 6.2). Summing up, it seems that there is a tendency in the reconstructed phylogenetic groups to relate with soil characteristics (Fig. 2a).

*T.fanfani* showed a larger range of soil textures and narrow pH soil preferences (Table 1) and is always associated with *T.guttata*. No differences in soil pH ranges can be linked to the intraspecific groups observed in the reconstructed phylogeny. However, soil texture preferences are slightly different in both clades, with one group including 65% specimens collected in sandy soils (lSs and Ss) and the other group with a higher preference value (77%) for this type of soils. Overall, *T.fanfani* seems to prefer slightly,to strongly, acid soils and, as for *T.arenaria*, a diversity linkage to sandier or loamier soil preferences is suggested (Fig. 2b). *T.extremadurensis* occurs in strongly to moderate acid loamy soils (Table 1), mainly with *T.guttata* (only in one Spanish record, GenBank HQ698134) *Cistusalbidus* is considered as putative host). The first group integrates specimens collected in the same region and no pattern is apparent concerning soil features.

This is the first report of *T.grisea* in this region. More interesting, *T.grisea* was considered exclusively an alkaline soil species until the present work. We have shown *T.grisea* presence in moderately to slightly acidic soils, mainly sandy soils and in association to *T.guttata* (Table 1). The two reconstructed groups (Fig. 2c) suggest a separation between variants, one associated with alkaline soils and hosted by *Pinus* spp. and the other associated with acid soils and linked to *T.guttata*. This separation is not clear-cut, however, as two samples collected in Burgos (Spain; GenBank KP189328 and GenBank KP189333) are nested in separate groups and are reported as collected in alkaline soil and on *Helianthemum* sp. host.

*T.lusitanica* occurs in strongly to slightly acidic sandy soils exclusively with *T.guttata* (Table 1). The reconstructed intraspecific phylogeny suggests three well supported groups, albeit with few representatives (Fig. 2d). The group with the highest number of specimens were mainly collected in lSs (72%) with a wide pH range (5.5 to 6.2), which separates them from the rest of the specimens, which were collected in Ss and at the higher range of the soil pH scale registered for this species (6.1). A single specimen was encountered on Ss at lower pH.

*T.pini* occurs in strongly to moderate acid loamy soils associated with *Quercus* spp. and *Pinus* spp (Table 1). The two first reconstructed intraspecific groups (Fig. 1) integrate specimens associated with both *Quercus* and *Pinus*, the first with a pH range from 5.3 to 6.0 and the second in soils with the same pH value (5.4). The remaining groups comprise specimens collected in association with *Quercus*. No tendency is apparent on both putative hosts and soil pH that could be linked to the intraspecific variability observed.

The recently described *T.solaris-libera* occurs in moderate acid loamy soils associated with *T.guttata* (Table 1), and this is consistent for all specimens regardless of their geographic origin. *T.cistophila* occurs in strongly to moderate acid sandy soils associated with *Cistus* spp. (Table 1). This is consistent with all samples but one (GenBank KP728828), which does not group with the others and it is originated from Greece and associated with *C.monspeliensis* and *C.creticus* (and no information on soil type is available). The remaining specimens are from Portugal and Spain and are linked to *C.salviifolius* and *C.ladanifer*. *T.dunensis* is so far represented by four specimens and only one with soil features information (Table 1). The two samples from Huelva (Spain) cluster together and are linked to *Cistaceae*. The other two samples are the “unclassifiable” GenBank AF396864 (Louro et al. 2019) and a sample from South Portugal that is either associated with *Cistus* or *Pinus*.

**Figure 3.**
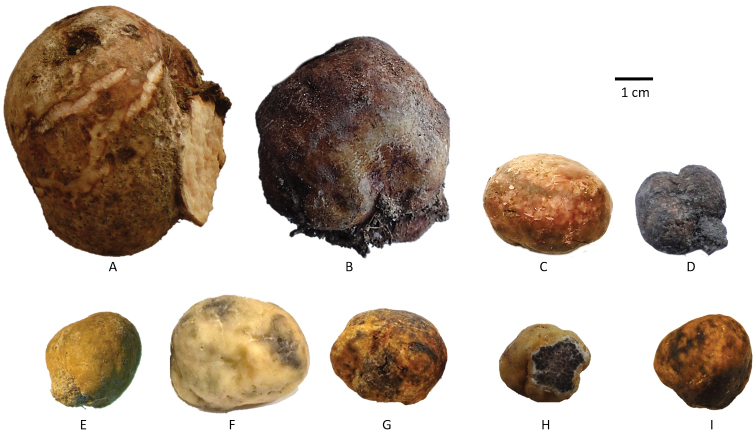
*Terfezia* species collected in the present work **A***T.arenaria***B***T.fanfani***C***T.cistophila***D***T.grisea***E***T.dunensis***F***T.extremadurensis***G***T.lusitanica***H***T.pini***I***T.solaris-libera*.

The nine *Terfezia* species collected in the present work are illustrated in Fig. 3.

## Discussion

The introduction of the newly collected *Terfezia* samples on the most recent published phylogenetic reconstruction of the genus (Louro et al. 2019) reinforces the existence of 18 well supported clades. Yet, GenBank AF396864, which at the time did not cluster with any of the other taxa, now fell under the newly described *T.dunensis* clade. This suggests that we might be closer to solving the identity of this previously unassigned sequenced if its position within the *T.dunensis* clade is to be sustained in subsequent studies with new data. The primary fungal barcode ITS remains the most informative DNA fragment available, and the great majority of available sequence data is based on this region. Although it is widely accepted as a standard molecular marker, some issues remain unresolved and other types of markers (e.g. microsatellites or, ideally genome wide data) might be needed to shed light on inter-species diversity and evolutionary patterns.

The comprehensive sampling along eight consecutive years, allowed us to update the existing knowledge on *Terfezia* species diversity in the region, and expand the number of species occurring in the country to 10 species (i.e. *T.alsheikhii*, *T.arenaria*, *T.cistophila*, *T.extremadurensis*, *T.fanfani*, *T.grisea*, *T.lusitanica*, *T.pini*, *T.olbiensis* and *T.solaris-libera* sp. nov.). Though *Terfeziaalsheikhii* was only registered once for Portugal (Bordallo et al. 2013), we were unable to find any specimen of this species and thus to confirm its presence. *Terfeziadunensis* and *T.grisea*, on the other hand, had never been registered in Portugal and therefore the present work represents the first record of their presence. The significance of these findings go beyond the scope of national or regional species checklist as they prove undoubtedly that the Iberian Peninsula, as a whole, is a diversity hotspot for the genus *Terfezia* given that every one of the eighteen accepted species occur in the territory. This documented outstanding diversity can be explained by the abundance of different putative hosts occurring on the Peninsula, as host specialization and edaphic tolerances likely played significant roles in *Terfezia* adaptive evolution (Diez et al. 2002; Bordallo and Rodríguez 2004).

More importantly, the present work examined the observed intraspecific variability within the context of soil and host preferences. The here achieved better understanding of the edaphic preference and host specificity of the analyzed *Terfezia* species is of the utmost importance in the framework of desert truffle cultivation. Although we found that the sampling area was not an influencing factor to explain the multiple lineages seen within each clade, we were able to identify some tendencies linking different *Terfezia* lineages within species to ecologic parameters such as soil texture, soil pH and host plant.

As such, the finding of *T.grisea* in acid soils is puzzling and contradicts the original species description. Our reconstructed phylogeny suggests a separation between two variants, one associated with alkaline soils and hosted by *Pinus* spp. and the other with acid soils and linked to *T.guttata*. Yet, this separation is not clear-cut, since the two existing Spanish sequences associated with *Helianthemum* spp. were represented in both sub-clades. Further sampling of this species is still needed in order to clarify if this clade represents a group of cryptic species, a single species that is undergoing speciation or a single species that has a wide edaphic tolerance and low host specificity.

The other two species with clear intra-species variability are *Terfeziaarenaria* and *T.fanfani*, both associated with a higher number of samples. These two species seem to be much more abundant but are also much more conspicuous because of their size. Whether the observed intra-species diversity can be linked to clear ecological preferences remains unknown. For *T.arenaria* we could observe a grouping tendency based on pH and soil type tolerance. For *T.fanfani*, differential preferences were also observed on these variables, albeit less defined. In both cases, the intra-specific diversity found in these species calls for a more detailed study including a set of meaningful ecological variables, forest and land management options. Concerning the last, it is reported that macrofungal richness, particularly for mycorrhizal *taxa*, are shaped by tree canopy density (Santos-Silva et al. 2011) and negatively affected by severe soil tillage and intensive grazing (Santos-Silva and Louro 2016; Pinto-Correia et al. 2018).

Understanding *Terfezia* diversity and its ecological constraints is highly relevant when considering the economic value of the desert truffles for rural populations on the Mediterranean basin. Desert truffles are a potentially important food source that is highly valued in local markets. A shift from expert collector to cultivation would enhance the socio-economic development of rural and/or local populations. To efficiently mass produce *Terfezia* one needs to explore the best genotype-host species combination but also learn the growing determinants that lead to a more efficient growth and fruitbodies production. *T.arenaria* and *T.fanfani* are by their abundance and size the most promising for cultivation purposes. In fact, most of the other *Terfezia* species have small size, do not fructify every year and are even harder to find. The attempt to describe its ecology is thus of upmost importance to confirm their identity, distribution and fructification patterns.

## Conclusion

The present work attempts, to the best of our knowledge for the first time, to systematically associate the diversity of *Terfezia* species with soil type, pH and with a putative host plant in a geographically limited sampling area. By doing so, it contributes to our knowledge of the species in the region, increasing the number of species to ten, opening the cultivation possibilities to other species, other host plants and to a wider range of soil types. To notice the first reference of *T.grisea* in acidic soils. No doubt *T.arenaria* and *T.fanfani* are the most found *Terfezia* species, either by their size, by their abundance or by a combination of both. We need to increase our knowledge on the crucial ecological determinants affecting desert truffles if we want to understand their diversity and cultivation potential.

## Supplementary materials

Supplementary material 1 Table S1 Data type Taxonomic, geographical and ecological information Explanation note This file discloses all *Terfezia* sequences falling within each clade on the phylogenetic analysis generated in this work (including the 45 sequences selected from the most recent genus phylogenetic reconstruction and the 216 newly generated sequences from this work) and their respective accession numbers and bibliographic references. A collection/sampling number is also provided for each one of the new sequences pertaining to the samples deposited at the Évora University Herbarium (UEVH-FUNGI). https://binary.pensoft.net/file/600007This dataset is made available under the Open Database License (http://opendatacommons.org/licenses/odbl/1.0/). The Open Database License (ODbL) is a license agreement intended to allow users to freely share, modify, and use this Dataset while maintaining this same freedom for others, provided that the original source and author(s) are credited.Celeste Santos-Silva, Rogério Louro, Bruno Natário, Tânia Nobre
